# Prevalence and management of hypertension among adults aged 45 and older in mainland China: Insights from the 2020 CHARLS survey

**DOI:** 10.1097/MD.0000000000047704

**Published:** 2026-02-20

**Authors:** Aiai Zhu, Yanna Le, Lei Zhang, Zhiwei Leng, Changfeng Hu, Chengjian Cao, Luoxian Yang

**Affiliations:** a Medical Education Department, Hangzhou Hospital for the Prevention and Treatment of Occupational Diseases, Hangzhou, Zhejiang, China; b College of Basic Medical, Peking Union Medical College, Beijing, China; c College of Basic Medical Sciences, Zhejiang Chinese Medical University, Hangzhou, Zhejiang, China.

**Keywords:** Chinese mainland, hypertension, prevalence, prevent and management, public health

## Abstract

Hypertension is an escalating global public health challenge. This study aimed to assess the prevalence, awareness, treatment, and management of hypertension, particularly the role of primary healthcare facilities, in individuals aged ≥ 45 years in mainland China. Data were derived from the 2020 baseline survey of the China Health and Retirement Longitudinal Study, comprising 19,282 participants. Statistical analyses were performed using SPSS Statistics 25.0 (Chicago), to examine hypertension prevalence across different demographic subgroups, along with detection rates, treatment status, and utilization of medical and health services. The overall prevalence of hypertension in the study population was 37.73%, with rates increasing with age and peaking in the 85 to 94 age group. While no statistically significant differences were found by gender, region, or education level, prevalence was markedly higher among individuals of the Uygur, Tibetan, and Mongolian ethnicities. Divorced or widowed individuals exhibited significantly higher hypertension rates than those of other marital statuses. Notably, 27.25% of hypertensive individuals were diagnosed through routine physical examination. Regarding disease management, 78.9% of hypertensive patients reported using one or more antihypertensive medications, and 77.9% believed that their blood pressure was well controlled. On average, 72.6% of patients received blood pressure monitoring services at primary healthcare facilities approximately 11.91 ± 4.36 times per year. However, <50% of the patients reported receiving health education for the prevention of hypertension-related complications. Hypertension is highly prevalent among middle-aged and older adults in Mainland China. Enhancing the effectiveness of primary healthcare services is essential to improve the diagnosis and treatment of hypertension. Greater efforts are needed to strengthen health education, raise public awareness, and promote self-management among patients to reduce the burden of hypertension and its associated complications.

## 1. Introduction

Hypertension, traditionally defined as a persistent office blood pressure of ≥140/90 mm Hg, is recognized as the leading modifiable risk factor for global cardiovascular diseases (CVDs) and related disability.^[1]^ Currently, it is the most prevalent chronic condition worldwide, affecting over 1 billion individuals,^[2]^ and its global burden continues to rise. Despite substantial advances in the understanding of hypertension prevention and treatment, its incidence, prevalence, and cardiovascular complications remain largely uncontrolled, particularly among aging populations, owing to persistent gaps in prevention, diagnosis, and management.^[3]^ As global life expectancy increases and individuals face greater exposure to cardiovascular risk factors, hypertension and its associated health consequences are drawing increasing public health attention.

Hypertension has emerged as a significant public health concern in China.^[4]^ In recent years, the country has experienced rapid population aging, lifestyle changes, and shifts in the chronic disease landscape, all contributing to a continuous rise in the incidence of hypertension, now increasingly affecting younger age groups.^[5]^ The prevalence of hypertension increases with age, rising from approximately 27% in individuals aged <60 years to 74% in those aged >80.^[6]^ Evidence from the Framingham Heart Study further suggests that more than 90% of individuals with normal blood pressure at the age of 55 eventually develop hypertension in later life.^[7]^ Projections indicate that by 2035, over 30% of China population will be aged 60 years or older.^[8]^ Consequently, the burden of hypertension is anticipated to grow substantially alongside the demographic shift toward an aging society. Given that hypertension is the leading modifiable risk factor for CVDs, implementing effective strategies for its prevention and management could significantly reduce the national healthcare and economic burden. Therefore, a comprehensive understanding of the current status of hypertension, particularly among the older adults population in mainland China, is essential.

Moreover, the management of hypertension in older adults presents numerous challenges,^[9]^ including a lack of consensus on optimal blood pressure targets, complexities in diagnosis and monitoring, and the need to weigh treatment benefits against potential adverse effects,^[10]^ and lack of effective management measures for self-monitoring of blood pressure^[11]^ and medication adherence.^[12]^ Although previous survey data from 2015 indicated a markedly higher prevalence of hypertension among middle-aged and older individuals compared to younger age groups, limited information is available regarding the current status of hypertension prevention and management in this demographic in mainland China.^[13]^

By leveraging baseline data from the 2020 China Health and Retirement Longitudinal Study (CHARLS), the present study aimed to investigate the prevalence, treatment, and control rates of hypertension among individuals aged 45 years and older. Furthermore, it evaluated the role of primary medical and healthcare facilities in the prevention and management of hypertension. By addressing these aspects, this study seeks to provide a clearer understanding of the hypertension landscape in China aging population and to inform targeted strategies for improving hypertension control and public health outcomes.

## 2. Material and methods

### 2.1. Data source and study design

The data for this study were obtained from the CHARLS, a nationally representative survey conducted by the National Development Research Institute of Peking University. CHARLS targets middle-aged and older adults in China and employs a multistage probability proportional to size sampling method. Through this approach, approximately 20,000 individuals aged 45 years and above were recruited across the country. The baseline national survey covered 450 village-level sampling units across 150 districts and counties across 28 provinces.

All survey protocols were approved by the Institutional Review Board of the Ethics Committee of Peking University. Written informed consent was obtained from all participants prior to commencement of data collection. Data were gathered through in-person interviews using standardized questionnaires and medical examinations. The survey covered a wide range of topics, including demographic and sociological characteristics, socioeconomic status, self-reported health, and physical functioning.

**Figure 1.**
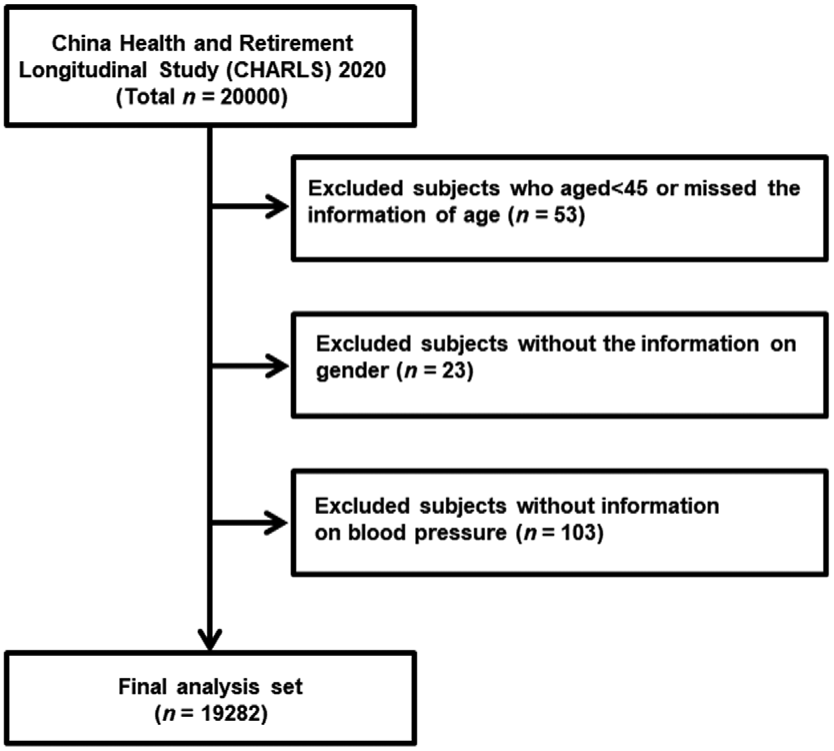
Flow chart of selecting study subjects from the CHARLS 2020 data questionnaire. CHARLS = China Health and Retirement Longitudinal Study.

Data from the 2020 CHARLS wave were used for the current analysis. Individuals under the age of 45 as well as those missing critical information such as gender, age, or blood pressure measurements were excluded. Only participants with complete demographic and sociological variables, family structure, household communication, socioeconomic indicators, self-reported health status, and functioning were included. After applying these criteria, 19,282 of the initial 20,000 respondents were deemed eligible for the analysis (Fig. 1).

### 2.2. Definition of hypertension components

This study used 2 sections of the CHARLS 2020 questionnaire. The first section included respondents’ basic demographic information such as gender, age, marital status, ethnicity, and place of residence. In this study, hypertension was defined as a clinical condition characterized by elevated systemic arterial blood pressure, systolic blood pressure ≥140 mm Hg, and/or diastolic blood pressure ≥90 mm Hg. The identification of hypertension was based on a physician’s diagnosis, recorded in the respondents’ medical records, and confirmed by at least 3 separate blood pressure measurements taken on different days. The prevalence of hypertension was defined as the proportion of individuals diagnosed with hypertension in the study population. The second section focused on hypertension management, particularly blood pressure measurements conducted by community healthcare providers. Community-based blood pressure monitoring was defined as blood pressure readings obtained either through self-service electronic monitors or with the assistance of community physicians. The hypertension control rate was defined as the proportion of individuals with hypertension who were able to maintain their blood pressure within the normal range through pharmacological treatment.

In this study, the presence of chronic diseases, specifically hypertension, was treated as a dependent variable. The independent variables included sex, marital status, and ethnicity. These variables were used to assess the prevalence of hypertension across the various demographic subgroups. Additionally, the analysis examined medication usage among individuals diagnosed with hypertension, their hypertension control status, and the availability of blood pressure monitoring services provided by primary healthcare facilities. This included factors such as the frequency of measurements, associated costs, and delivery of relevant health education.

### 2.3. Other variables

All survey participants were stratified into 2 primary subgroups and 6 age brackets: 45 to 54, 55 to 64, 65 to 74, 75 to 84, 85 to 94, and ≥95-years old. The first 2 age groups predominantly comprised individuals within the workforce, whereas the latter 4 represented the retired older adults population. Based on marital status, participants were categorized as married or cohabiting with a spouse, married but not currently living with a spouse, separated (no longer cohabiting as spouses), divorced, widowed, or never married. The ethnic classification included Han Chinese, Zhuang, Manchu, Hui, Miao, Uyghur, Tujia, Yi, Mongolian, Tibetan, and other ethnic minorities (those with fewer than 100 individuals were consolidated into a single “other minorities” group). Additionally, participants were categorized by their place of residence into 4 groups: city/town, suburban, rural village, and special areas.

### 2.4. Statistical analysis

Statistical analyses were performed using SPSS version 25.0 (Chicago). To ensure the national representativeness of the prevalence estimates, a complex sampling design and sampling weights were applied, incorporating nonresponse-adjusted individual sample weights. The chi-square (χ^2^) test was used to compare prevalence rates across different population subgroups and to evaluate the associations between categorical variables (expressed as frequencies and percentages) and hypertension prevalence, control rates, and utilization of community health services. Statistical significance was set at *P* <.05.

The reliability and validity of the CHARLS 2020 questionnaire were also assessed. Cronbach alpha coefficient was 0.63, indicating acceptable internal consistency. The KMO coefficient was 0.72, and Bartlett test of sphericity yielded a *P*-value of .04 (less than .05), confirming that the questionnaire demonstrated acceptable construct validity.

## 3. Results

### 3.1. Demographic backgrounds

A total of 19,282 Chinese individuals aged >45 years participated in the survey, including 9190 males and 10,092 females. Participants ranged in age from 45 to 111 years, with a mean age of 64.91 ± 10.19 years. The age distribution was as follows: 3271 individuals aged 45 to 54 years (16.97%), 6359 aged 55 to 64 years (32.98%), 6211 aged 65 to 74 years (32.21%), 2666 aged 75 to 84 years (13.83%), 715 aged 85 to 94 years (3.71%), and 59 aged ≥95 years (0.31%).

Regarding marital status, 15,162 participants (78.63%) were married and living with their spouse, 1290 (6.69%) were married but not living with their spouse temporarily (e.g., due to work), 69 (0.36%) were separated, 235 (1.22%) were divorced, 2415 (12.52%) were widowed, and 111 (0.58%) had never married.

In terms of residence, 14,301 participants (73.07%) lived in villages, 3513 (17.95%) lived in city or town centers, 1390 (7.11%) lived in suburban or urban-rural transitional areas, and 78 (0.40%) lived in designated special regions (Table 1).

**Table 1:** Basic information and prevalence of hypertension among survey subjects.

Grouping	Groups	Number of people	Proportion of population (%)	Number of people with hypertension	Prevalence of hypertension within the group (%)	Comparison of differences between groups	
						χ^2^	*P*
Gender	Male	9190	47.66	3443	37.46	0.930	.269
	Female	10,092	52.34	3833	37.98	–	–
Age	45–54	3272	16.97	680	20.78	1010.783	<.001
	55–64	6359	32.98	2061	32.41	–	–
	65–74	6211	32.21	2700	43.47	–	–
	75–84	2666	13.83	1420	53.26	–	–
	85–94	715	3.71	387	54.13	–	–
	≥95	59	0.31	28	47.46	–	–
Marriage status	Married and living together with spouse	15,162	78.63	5562	36.68	200.621	<.001
	Married, but temporarily not living with spouse due to work or other reasons	1290	6.69	365	28.29	–	–
	Separation, no longer living together as a spouse	69	0.36	23	33.33	–	–
	Divorced	235	1.22	89	37.87	–	–
	Widowed	2415	12.52	1197	49.57	–	–
	Never married	111	0.58	40	36.04	–	–
Nation	Han Chinese	17,780	92.21	6653	37.42	26.553	.240
	Manchu	314	1.63	132	42.04	–	–
	Mongols	210	1.09	105	50.00	–	–
	Other ethnic minorities	978	5.66	386	39.47	–	–
	The center of city/town	3513	17.95	1317	37.49	2.518	.472
Address	Combination zone between urban and rural areas	1390	7.11%	520	37.41	–	–
	Village	14,301	73.07	5403	37.78	–	–
	Special area	78	0.40	36	46.15	–	–
Overall rate	–	19,282	100	–	–	–	–

### 3.2. Prevalence of hypertension

Among Chinese individuals aged over 45 years, 7276 participants were identified as having hypertension, yielding an overall prevalence rate of 40.83%. Of these, 3443 were men (prevalence rate: 40.25%) and 3833 were women (prevalence rate: 41.36%), with no statistically significant difference between the sexes (χ^2^ = 0.930, *P* = .269).

A statistically significant difference in hypertension prevalence was observed across the age groups (χ^2^ = 1010.783, *P* <.001). The first 2 age groups (45–54 and 55–64 years), which primarily comprised the working population, exhibited lower prevalence rates than the older groups. The prevalence increased progressively with age from 45 to 94 years, peaking in the 85 to 94 years age group at 54.13% (387/715).

There was no difference in the prevalence of hypertension between those living in China and those living abroad (χ2 = 0.008, *P *= .629). Marital status was found to significantly influence the prevalence of hypertension (χ^2^ = 200.621, *P* <.001), with the widowed group exhibiting the highest prevalence rate of 49.57%.

No statistically significant difference in hypertension prevalence was observed among the different ethnic groups (χ^2^ = 26.553, *P* = .240). Similarly, no significant differences were found across different residential categories. Detailed results are presented in Table 1.

### 3.3. Hypertension control rate

Statistically significant differences were observed in blood pressure control rates among hypertensive patients of different sexes. Male patients exhibited a higher control rate (25.01%) than female patients (21.89%) (χ^2^ = 7276.000, *P* <.001).

There were also significant differences in the control rates across age groups (χ^2^ = 101.297, *P* <.001). The highest blood pressure control rate was found in the 45 to 54-year-old group (36.18%), followed by the 55 to 64-year-old group (26.15%), both predominantly composed of individuals still in the workforce. In contrast, the group aged ≥95 years had the lowest control rate.

Marital status significantly influenced hypertension control rates (χ^2^ = 23.627, *P* <.001). The highest control rate was observed in the group “Married but not currently living with their spouse due to work or other reasons” (32.33%), followed by the group “Married and living with their spouse” (23.28%). The lowest control rates were seen in the “Widowed” group (20.72%) and the “Never married” Never-married group (20.00%).

No statistically significant differences in hypertension control rates were observed among the different ethnic groups (χ^2^ = 314.223, *P* = .371) or among participants residing in different geographic locations (χ^2^ = 1.174, *P* = .759) (Table 2).

**Table 2:** The blood pressure control rate among hypertension subjects.

Grouping	Group	Number of people with hypertension	Number of hypertension whose blood pressure was under control	Blood pressure control rate (%)	Comparison of differences between groups	
					χ^2^	*P*
Gender	Male	3443	861	25.01	7276.000	<.001
	Female	3833	839	21.89	–	–
Age (years)	45–54	680	246	36.18	101.297	<.001
	55–64	2061	539	26.15	–	
	65–74	2700	573	21.22	–	
	75–84	1420	266	18.73	–	
	85–94	387	70	18.09	–	
	≥ 95	28	6	21.43	–	
Marital status	Married and living together with spouse	5562	1295	23.28	23.627	<.001
	Married, but temporarily not living with spouse due to work or other reasons	365	118	32.33	–	
	Separation, no longer living together as a spouse	23	5	21.74	–	
	Divorced	89	26	29.21	–	
	Widowed	1197	248	20.72	–	
	Never married	40	8	20.00	–	
Nation	Han Chinese	6653	1575	23.67	314.223	.371
	Manchu ethnic group	132	21	15.91	–	
	Mongols	105	24	22.86	–	
	Other ethnic minorities	315	63	20.00	–	
Address	The center of city/town	1317	297	22.55	1.174	.759
	Combination zone between urban and rural areas	520	118	22.69	–	
	Village	5403	1275	23.60	–	
	Special area	36	10	27.78	–	
Overall rate	–	7276	1700	23.36	–	–

### 3.4. Community prevention and treatment of hypertension

Among the hypertensive patients surveyed, 5133 did not respond to the question regarding how their hypertension was detected. Among those who responded, 734 patients (34.25%) reported being diagnosed during a “post-seizure examination,” 540 (25.20%) after seeking medical care due to illness, and 127 (5.26%) during a “physical examination organized by their workplace.” Additionally, 397 individuals (18.53%) indicated that their hypertension was identified through community-organized physical examinations, and 60 (2.80%) were diagnosed via CHARLS physical examination. In total, 584 patients (27.25%) reported that hypertension was detected through routine or preventive physical examinations. Moreover, 285 individuals (13.30%) were diagnosed using other unspecified methods.

Among hypertensive patients who received medical services from primary medical and health facilities (community or village doctors), 5282 (80.43% of the total) reported having their blood pressure measured within the past year. Among these patients, the average frequency of blood pressure measurements was 11.91 ± 4.36 times per year, with 96.64% of the services provided free of charge. Regarding the frequency of blood pressure monitoring by community doctors, 485 patients (9.18%) reported measurements “once a week,” 626 (11.85%) “once every half month,” 1361 (25.77%) “once a month,” 613 (11.61%) “once every 2 months,” 702 (13.29%) “once a quarter,” 767 (14.52%) “once every half year,” and 726 (13.74%) “once a year.”

When asked about recommendations from community doctors for blood pressure control, hypertensive patients reported receiving the following advice: 2228 patients (30.62%) were advised to control their weight, 2945 (40.48%) were encouraged to increase physical activity, 3391 (46.61%) received dietary guidance, and 1880 (25.84%) were advised to quit smoking. Despite this, 246 patients (13.09% of those identified as needing to quit smoking) continued smoking. Notably, 2918 patients (40.10%) reported receiving no guidance on blood pressure management by community doctors.

When asked about lifestyle habits, 344 hypertensive patients (4.73%) reported current smoking, whereas 5016 (68.94%) reported alcohol consumption.

Regarding blood pressure management, 5725 patients (78.68%) believed that their blood pressure was under control. At the time of the survey, 5654 patients (77.71%) were taking antihypertensive medications. Among them, 364 (6.44%) were using traditional Chinese medicine, 5083 (89.90%) were taking Western medicine, and 207 (3.66%) reported using other types of medications for blood pressure reduction.

## 4. Discussion

The study data were collected from CHARLS, which is recognized for its rigorous research design and strict quality control. While several hypertension-related data have been utilized in published studies in China,^[14–19]^ questions related to patients’ perspectives on basic-level medical facilities (DA011-14) were not utilized in the questionnaire. However, these surveys could provide valuable insights into hypertension prevention and treatment in China basic-level medical facilities in conjunction with chronic disease prevention and control norms.

Data analysis revealed that the prevalence of hypertension among people aged 45 years and above in China was 37.73%. No statistically significant difference in hypertension incidence was observed among the different sex and place of residence groups. However, variations in hypertension rates were noted among different age groups and marital statuses. With increasing age, hypertension rates initially increased, reaching the highest levels in the 85 to 94 age bracket, although the 95-years old group showed a decrease, likely because of the relatively low number of investigations in this group. The prevalence of hypertension was highest in the widowed group (49.57%), followed by the divorced group (37.87%), and married and living with spouse (36.68%), suggesting that divorce and widowhood were associated with higher hypertension rates. Furthermore, statistical differences in the prevalence of hypertension were found among different ethnic groups and religious beliefs, potentially linked to diverse eating habits and lifestyles.

The data analysis also revealed that the hypertension control rate among people aged 45 years and above in China was 23.36%. No statistically significant difference in blood pressure control rate was observed among hypertensive patients of different ethnic groups and residential areas. However, differences in the hypertension incidence rate were identified among different sexes, age groups, and marital status. The blood pressure control rate of male hypertensive patients was higher than that of female patients, suggesting a gender-based discrepancy in blood pressure control. Similarly, as with hypertension prevalence, blood pressure control rates demonstrated an upward trend with age, reaching its peak among people aged 85 to 94, subsequently decreasing in the 95-year-old group, indicating age as a negative factor for hypertension control. Among different marital statuses, “Married, but temporarily not living with spouse due to work or other reasons” group had the highest blood pressure control rate at 32.33%, followed by the Divorced group at 29.21%. The 2 groups with the lowest blood pressure control rates are the Widowed group and the “never married” group, which may be related to the relatively single dietary habits and lifestyle of patients with these 2 marital statuses, as well as the lack of family support and supervision from other family members. This is consistent with the previously reported.^[20]^ A review of the relevant literature and reports revealed that the living arrangements of diabetic patients may affect the effectiveness of blood sugar and blood pressure management in controlling cardiovascular disease. Compared with diabetic patients living alone, those not living alone had better control over their blood sugar and blood pressure, which is consistent with the findings of this study.^[21]^

Data analysis indicated a relatively low rate of hypertension detection during routine and preventive physical examinations, with only 27.25% of the patients identified. “Post attack” and “Post illness” evaluations demonstrated relatively high proportions at 33.3% and 24.4%, respectively, indicating concealed onset of hypertension discovered only after symptoms or concurrent cardiovascular and cerebrovascular diseases. This underlines the need for improved early detection measures as outlined in the National Norms for the Prevention and Control of Chronic Diseases^[22]^ Suggestions for enhancing detection include the incorporation of automatic or semi-automatic electronic sphygmomanometers in public spaces to facilitate blood pressure measurements and elevate detection probabilities.^[23]^ Although 78.68% of patients believed that their blood pressure was under control, while 77.71% of hypertensive patients reported using medication to control their blood pressure, the self-perceived blood pressure control rate aligns closely with the medication rate. The main antihypertensive treatment strategy involved Western medicine (89.90%) supplemented by Chinese medicine (6.44%). Notably, medication adherence significantly affected the pressure control. With nearly 1/5 of patients (the medication rate is only 77.71%) not using antihypertensive drugs, there was a spotlight on the importance of reinforcing understanding of medication compliance among hypertensive patients. Improving medication compliance among patients with hypertension is also a key focus of hypertension prevention and treatment in primary healthcare facilities.

80.43 Of the patients, 80.43% received blood pressure measurements from doctors in grassroots medical facilities, a significantly higher proportion than those diagnosed with hypertension during routine and preventive physical examinations (27.25%). This indicates that many hypertensive patients prioritized routine measurements in grassroots medical facilities post-diagnosis. On average, blood pressure was measured 11.91 times per year at grassroots medical and health facilities, meeting chronic disease prevention and control standards. Community doctors provided free blood pressure testing services, signifying that, overall, medical services for blood pressure in community health services were well-established. This underscored the crucial role played by grassroots medical facilities in controlling blood pressure and identifying complications in patients with hypertension. Given the long-term medication needs of patients with hypertension, regular blood pressure monitoring during medication administration could facilitate timely adjustments for optimal blood pressure control.

For individuals with hypertension, attention to blood pressure control is crucial.^[19,24]^ Given that the probability of receiving health education on key factors, such as weight control, physical exercise, dietary adjustment, and smoking control, was <50%, with 40.10% of patients reporting no advice received, it was apparent that there was a significant gap in health education provision for these patients. Even among hypertensive patients who are aware of the need to quit smoking, 13.09% continue to smoke, with low compliance; at the same time, a relatively high proportion (68.94%) of hypertensive patients currently have a drinking habit, which needs to be taken seriously by medical personnel. Health education methods for blood pressure control,^[25]^ while being separately promoted by medical staff, might lack tailored approaches, innovative activities, and resident engagement, resulting in limited effectiveness. It is recommended that medical personnel in grassroots medical facilities customize health education content for patients with hypertension, highlighting specific patient profiles and allowing patients to choose appropriate control measures tailored to their needs.^[26,27]^ This approach would enable patients to engage with and adopt suitable blood pressure control strategies.

This study was based on the CHARLS project, which involves a large number of survey subjects from various provinces and cities and can well represent the situation of middle-aged and older adults aged 45 and above in China. However, owing to the large number of survey contents and the fact that the theme is not only hypertension, the questionnaire design questions may not consider a comprehensive range of factors compared to survey projects that focus solely on hypertension. This is a limitation of the present study. Although the sample size was large, it was difficult to conduct a more detailed analysis of health. However, this can be supplemented by horizontal integration with other studies.

### 4.1. Strengths and limitations of this study

1. This study utilized nationally representative baseline survey data from the 2020 CHARLS to provide robust insights into the prevalence and management of hypertension among middle-aged and older adults in mainland China.
2. The analysis explored hypertension epidemiology across multiple sociodemographic dimensions, including sex, age, and marital status, to uncover population-level disparities in disease burden.
3. This study assessed the role of primary healthcare facilities in hypertension prevention and management by evaluating patients’ diagnostic pathways, blood pressure monitoring practices, and medication adherence, offering evidence-based suggestions for future policy improvements.
4. Given that CHARLS primarily focuses on aging-related issues, the available data on health determinants are limited, constraining further in-depth analysis of the biomedical, behavioral, and environmental factors influencing hypertension.

## 5. Conclusion

China 3-level hypertension prevention network relies on grassroots facilities and medical staff.^[28,29]^ To improve control among people aged 45 + years, efforts should focus on routine and preventive physical examinations, regular monitoring, and targeted interventions for high-risk groups such as the older adults, widowed/divorced individuals, and certain ethnic populations. Strengthening health education, addressing alcohol use, and enhancing medication adherence can improve hypertension management and reduce complications, thereby improving patients’ quality of life.

## Author contributions

**Methodology:** Aiai Zhu.

**Writing – original draft:** Aiai Zhu, Yanna Le, Lei Zhang, Zhiwei Leng, Changfeng Hu, Chengjian Cao, Luoxian Yang.

**Writing – review & editing:** Aiai Zhu.

## References

[1] Olsen MH Angell SY Asma S. A call to action and a lifecourse strategy to address the global burden of raised blood pressure on current and future generations: the lancet commission on hypertension. Lancet. 2016;388:2665–712. 27671667 10.1016/S0140-6736(16)31134-5

[2] Forouzanfar MH Alexander L Anderson HR; GBD 2013 Risk Factors Collaborators. Global, regional, and national comparative risk assessment of 79 behavioural, environmental and occupational, and metabolic risks or clusters of risks in 188 countries, 1990-2013: a systematic analysis for the Global Burden of Disease Study 2013. Lancet. 2015;386:2287–323. 26364544 10.1016/S0140-6736(15)00128-2 PMC4685753

[3] Roth GA Mensah GA Johnson CO; GBD-NHLBI-JACC Global Burden of Cardiovascular Diseases Writing Group. Global burden of cardiovascular diseases and risk factors, 1990-2019: update from the GBD 2019 study. J Am Coll Cardiol. 2020;76:2982–3021. 33309175 10.1016/j.jacc.2020.11.010 PMC7755038

[4] Cao X Wang X Tian YX. Trends and sociodemographic patterns in hypertension prevalence and treatment in China. Med Cell Press J. 2025;6:100808. 10.1016/j.medj.2025.100808 40865522

[5] Lu J Lu Y Wang X. Prevalence, awareness, treatment, and control of hypertension in China: data from 1.7 million adults in a population-based screening study (China PEACE Million Persons Project). Lancet. 2017;390:2549–58. 29102084 10.1016/S0140-6736(17)32478-9

[6] Lloyd-Jones DM Evans JC Levy D. Hypertension in adults across the age spectrum: current outcomes and control in the community. JAMA. 2005;294:466–72. 16046653 10.1001/jama.294.4.466

[7] Franklin SS Larson MG Khan SA. Does the relation of blood pressure to coronary heart disease risk change with aging? The Framingham Heart Study. Circulation. 2001;103:1245–9. 11238268 10.1161/01.cir.103.9.1245

[8] Yin R Yin L Li L. Hypertension in China: burdens, guidelines and policy responses: a state-of-the-art review. J Hum Hypertens. 2021;36:126–34. 34215840 10.1038/s41371-021-00570-z PMC8252986

[9] Lu XL Wang JW Chen SK Lv L Yu J. Effect of comprehensive health management on medication adherence and healthy lifestyle behavior of patients with hypertension. Int J Hypertens. 2025;2025:e11658097. 10.1155/ijhy/1165809 PMC12463532 41018530

[10] Oliveros E Patel H Kyung S. Hypertension in older adults: assessment, management, and challenges. Clin Cardiol. 2019;43:99–107. 31825114 10.1002/clc.23303 PMC7021657

[11] Yu YW He L Feng ZX. Optimizing hypertension management in China: care cascade insights and individual determinants from a national survey. Curr Med Res Opin. 2025;41:741–51. 40471694 10.1080/03007995.2025.2502674 PMC12312749

[12] Lu XL Wang JW Chen SK Lv L Yu J. Analysis of adherence status and influencing factors among middle-aged and elderly hypertension patients in rural areas of Northeast China. Int J Hypertens. 2025;2025:e9954099. 10.1155/ijhy/9954099 PMC12049249 40322455

[13] Joint Committee for Guideline Revision. 2018 Chinese guidelines for prevention and treatment of hypertension-A report of the revision committee of Chinese guidelines for prevention and treatment of hypertension. J Geriatr Cardiol. 2019;16:182–241. 31080465 10.11909/j.issn.1671-5411.2019.03.014 PMC6500570

[14] Wang H-y Meng Q Yang C. Association between pulse pressure, systolic blood pressure and the risk of rapid decline of kidney function among general population without hypertension: results from the China health and retirement longitudinal study (CHARLS). J Transl Med. 2021;19:512. 34930335 10.1186/s12967-021-03176-8 PMC8686555

[15] Qiu W Cai AA Li LL Feng Y. Association of depression trajectories and subsequent hypertension and cardiovascular disease: findings from the CHARLS cohort. J Hypertens. 2024;42:432–40. 37937504 10.1097/HJH.0000000000003609

[16] Lin L Wang HH Lu C Chen W Guo VY. Adverse childhood experiences and subsequent chronic diseases among middle-aged or older adults in China and associations with demographic and socioeconomic characteristics. JAMA Netw Open. 2021;4:e2130143. 34694390 10.1001/jamanetworkopen.2021.30143 PMC8546496

[17] Tang H Li M Liu LZ Zhou Y Liu X. Changing inequity in health service utilization and financial burden among patients with hypertension in China: evidence from China Health and Retirement Longitudinal Study (CHARLS), 2011–2018. Int J Equity Health. 2023;22:246. 38001484 10.1186/s12939-023-02062-7 PMC10668495

[18] Ding L Zhu X Xiong Z Yang F Zhang X. The association of age at diagnosis of hypertension with cognitive decline: the China Health and Retirement Longitudinal Study (CHARLS). J Gen Intern Med. 2022;38:1431–8. 36443629 10.1007/s11606-022-07951-1 PMC10160298

[19] Li Z Fu C Yang F Mao Z. Prevalence and risk factors of hypertension for the middle-aged population in China — results from the China Health and Retirement Longitudinal Study (CHARLS). Clin Exp Hypertens. 2018;41:80–6. 29553846 10.1080/10641963.2018.1445751

[20] Simegn BK Chelkeba L Alamirew BD. Clinicians’ prescribing pattern, rate of patients’ medication adherence and its determinants among adult hypertensive patients at Jimma university medical center: prospective cohort study. PLoS One. 2021;16:e0259421. 34780479 10.1371/journal.pone.0259421 PMC8592482

[21] Edward JA Josey K Bahn G. Heterogeneous treatment effects of intensive glycemic control on major adverse cardiovascular events in the ACCORD and VADT trials: a machine-learning analysis. Cardiovasc Diabetol. 2022;21:58. 35477454 10.1186/s12933-022-01496-7 PMC9047276

[22] Virgini V Meindl-Fridez C Battegay E Zimmerli LU. Check-up examination: recommendations in adults. Swiss Med Wkly. 2015;145:w14075. 25635626 10.4414/smw.2015.14075

[23] Chappell LC Tucker KL Galal U; BUMP 2 Investigators. Effect of self-monitoring of blood pressure on blood pressure control in pregnant individuals with chronic or gestational hypertension: the BUMP 2 randomized clinical trial. JAMA. 2022;327:1666–78. 35503345 10.1001/jama.2022.4726 PMC9066282

[24] Volpe M Gallo G Tocci G. Is early and fast blood pressure control important in hypertension management? Int J Cardiol. 2018;254:328–32. 29273242 10.1016/j.ijcard.2017.12.026

[25] Towfighi A Cheng EM Ayala-Rivera M; Secondary Stroke Prevention by Uniting Community and Chronic Care Model Teams Early to End Disparities (SUCCEED) Investigators. Effect of a coordinated community and chronic care model team intervention vs usual care on systolic blood pressure in patients with stroke or transient ischemic attack. JAMA Netw Open. 2021;4:e2036227. 33587132 10.1001/jamanetworkopen.2020.36227 PMC7885035

[26] Chobanian AV Bakris GL Black HR; Joint National Committee on Prevention, Detection, Evaluation, and Treatment of High Blood Pressure. National Heart, Lung, and Blood Institute. Seventh report of the Joint national committee on prevention, detection, evaluation, and treatment of high blood pressure. Hypertension. 2003;42:1206–52. 14656957 10.1161/01.HYP.0000107251.49515.c2

[27] Abdalla M Bolen SD Brettler J; American Heart Association and American Medical Association. Implementation strategies to improve blood pressure control in the United States: a scientific statement from the American Heart Association and American Medical Association. Hypertension. 2023;80:e143–57. 37650292 10.1161/HYP.0000000000000232 PMC10578150

[28] Sun Y Mu J Wang DW; CRHCP Study Group. A village doctor-led multifaceted intervention for blood pressure control in rural China: an open, cluster randomised trial. Lancet. 2022;399:1964–75. 35500594 10.1016/S0140-6736(22)00325-7

[29] Steyn K Fourie NL Rossouw K. Treatment status and experiences of hypertension patients at a large health center in Cape Town. Ethn Dis. 1999;9:441–50. 10600067

